# Penile fracture: An analysis of 9 cases in a tertiary hospital

**DOI:** 10.1016/j.amsu.2022.104028

**Published:** 2022-06-22

**Authors:** Maher Al-Hajjaj, Ali Alali Aljool, Hasan Al Husein

**Affiliations:** Department of Urology, Aleppo University Hospital, Aleppo, Syria

**Keywords:** Penile fracture, Penile ecchymosis, Urological emergency

## Abstract

**Introduction:**

Penile fracture (PF) is considered a rare condition that requires urgent surgical intervention. It is a result of rupture of the tunica albuginea of the corpus cavernosum.

**Materials and methods:**

We aimed to review the data of nine patients who presented to our hospital between May 2019 and May 2021 with penile fracture diagnosed clinically. Our patients underwent surgical repair. Age, etiology of penile fracture, clinical findings, and side of the defect were analyzed.

**Results:**

The mean age of the patients was 33.3 ± 10.5 years. The most common reason for penile fracture was sexual intercourse. 100% of our patients had penile ecchymosis. All patients underwent surgical exploration. None of them underwent retrograde urethrography. 55.5% of patients had right side defects. The defect was located proximally in six patients (66.6%)

**Conclusion:**

Penile fracture is an emergency case in urology. It could be diagnosed clinically most of the time. Early surgical repair can lead to fewer complications and good outcomes.

## Highlights

• Penile fracture is an uncommon presentation to Urology departments.
• It is a traumatic rupture of the tunica albuginea in the corpus cavernosum due to the rotation or bending of the penile shaft during erection.
• Audible “cracking” sound, immediate detumescence, pain, and swelling are the classic triad present in penile fracture.
• Current standard protocol for the treatment of fracture penis includes immediate surgical exploration of penis.

## 1 Introduction

Penile fracture (PF) is one of the important urological emergencies caused by strong manipulation, strong vaginal or anal sexual intercourse or masturbation, knife wounds, or any mechanical trauma that causes an erect penis to bend forcibly, causing a tear in the tunica albuginea of the penis [1].

The true incidence of penile fracture is much higher than reported because many patients do not seek medical attention due to embarrassment or fear [2].

The classic patient gives a history of hearing a cracking noise during sexual activity when the tunica ruptures, rapidly followed by pain, detumescence, and a substantial subcutaneous hematoma leading to an ‘eggplant deformity’ [3].

The current standard protocol for the treatment of fracture penis includes immediate surgical exploration of penis involving degloving of the penis, hematoma evacuation, and suturing of rent in tunica albuginea with nonabsorbable suture [4].

In this study, we aimed to analysis the clinical presentation, diagnosis, and management of nine cases of penile fracture presented to our emergency department.

This work has been reported in line with the PROCESS 2020 criteria [5].

## 2 Method

**Fig. 1:**
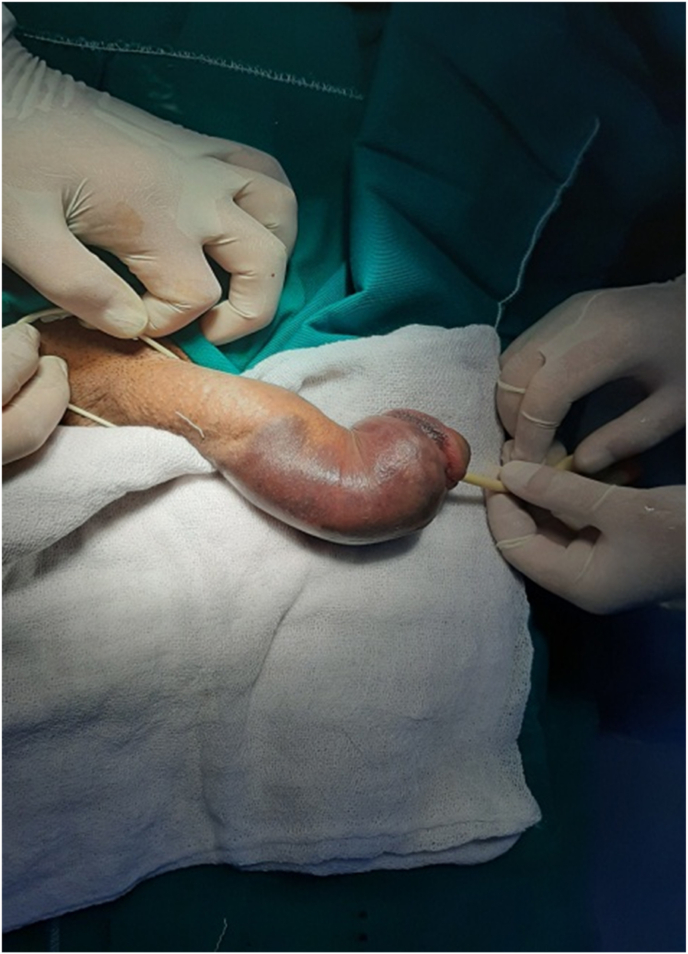
Penile fracture.

**Fig. 2:**
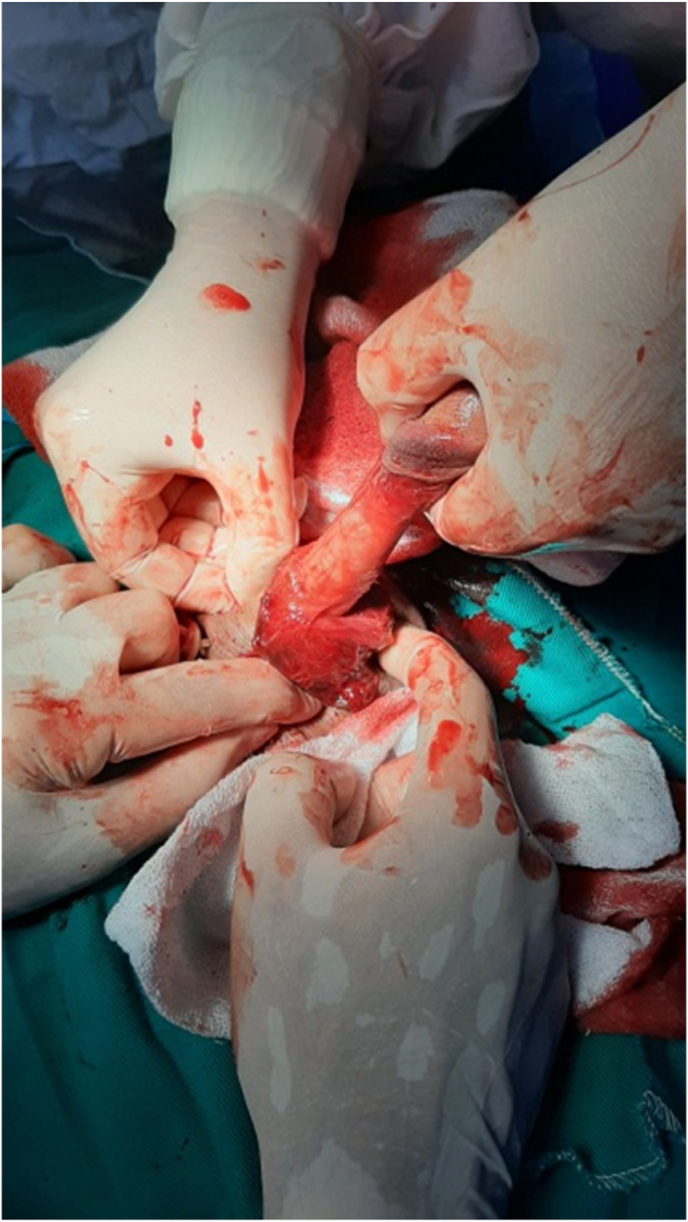
Degloving incision of penis.

**Fig. 3:**
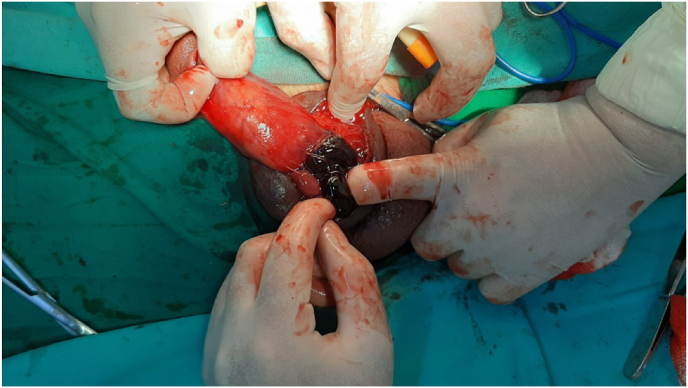
Case of fracture penis showing hematoma.

**Fig. 4:**
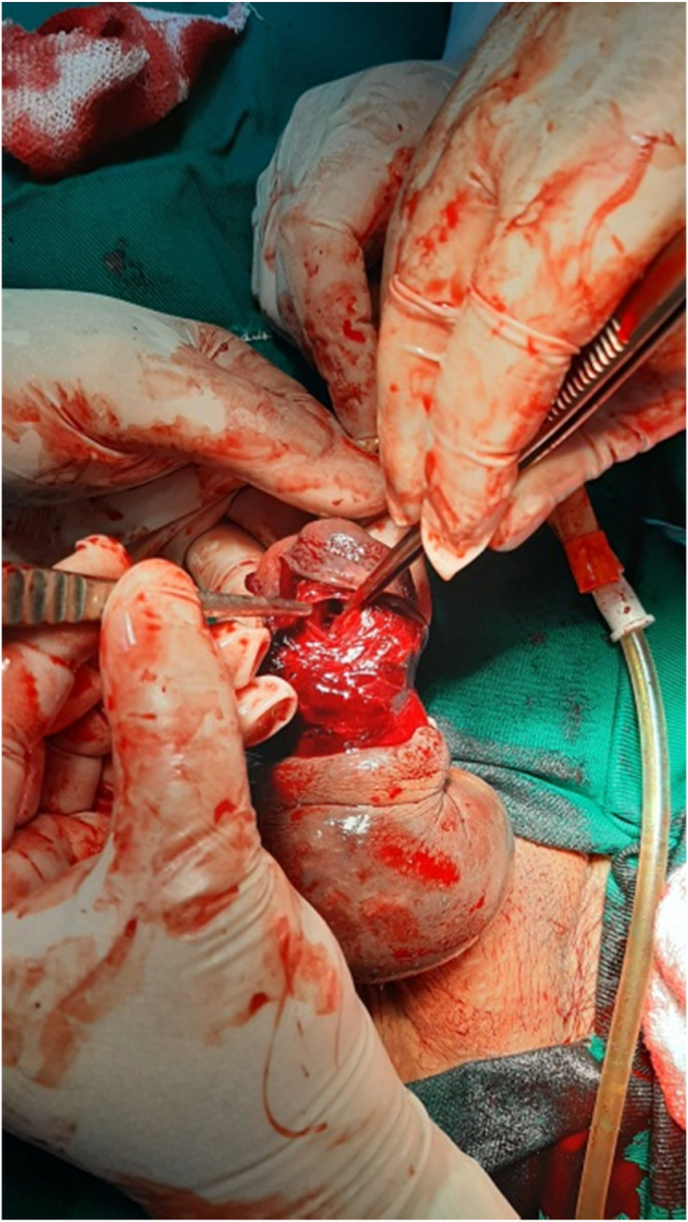
Intraoperative finding of cavernosal defect.

We conducted a retrospective study for two years between May 2019 and May 2021 at the department of urology, Aleppo University Hospital, Aleppo, Syria. We had nine patients with a penile fracture who were admitted to our hospital. Detailed history and good physical examination led to easy diagnosis of the penile fracture (Fig. 1). We did not use radiology images on any patients. All of our patients underwent surgical exploration. Informed written consent was obtained from patients before surgical exploration. We obtained written informed consent for surgery from all patients. Foley catheterization was done in all patients. A single dose of intravenous antibiotic prophylaxis before the intervention. We performed a Subcoronal circumferential incision. The penis was degloved to identify the exact location of the fracture in all the patients (Fig. 2). First, we removed the hematoma and we defined the exact place of the defect of the tunica albuginea (Fig. 3). Second, we repaired it by suturing it with 4/0 inverted absorbable sutures (Fig. 4).

An artificial erection with saline injection was performed to determine if there was penile curvature after fracture repair.

Catheters were removed on the third day of surgery in all patients.

## 3 Results

Our study included 9 cases of penile fracture. The mean age was 33.3 ± 10.5 years. The age distribution is shown in Table 1. Five patients (55.5%) had a penile fracture during sexual intercourse, three patients (33.3%) during masturbation and one patient (11.1%) had a penile fracture due to suddenly forced flexion (Table 2). Physical examination revealed 9 patients (100%) had penile ecchymosis, six patients (66.6%) had penile swelling, two patients (22.2%) had penile pain, eight patients (88.8%) had sudden detumescence, and six patients (66.6%) had sudden cracking noise (Table 3). Our patients were not undergone retrograde urethrography because none of them had urethral bleeding and urinary symptoms, or microscopic hematuria. The defect of the tunica albuginea was on the right side in five patients (55.5%), and four patients had it on the left side (44.4%) (see Table 4).

**Table 1:** Age of patients.

Age group	Number of patients
18–30	3
30–40	4
40–50	1
>50	1

**Table 2:** Causes of penile fracture.

Cause	Number of patients
Coitus	5
Masturbation	3
Sudden forced flexion	1

**Table 3:** Examination findings.

Clinical examination	Number of patients
Penile ecchymosis	9
Penile swelling	6
Penile pain	2
Sudden detumescence	8
Sudden cracking noise	6

**Table 4:** Site of tunica tear.

Site of fracture	Number of patient
Right corpus	5
Left corpus	4
Proximal shaft	6
Mid shaft	3

The defect was on the right side in five patients (55.5%), and on the left side in four patients (44.4%). The defect was located proximally in six patients (66.6%) and the mid shaft was in three patients (33.3%).

## 4 Discussion

The thickness of tunica albuginea is about 2 mm when the penis is not in an erect state, which decreases to 0.25–0.50 mm in the erection phase. Thus, it ruptures more easily if exposed to trauma [11]. While the average arterial pressure in the erect penis is around 100 mmHg, the pressure must be above 1500 mmHg for the corpus cavernosum rupture [6].

The usual mechanism of penile fracture is related to specific sexual activities that an individual engages in, masturbation, and socio-cultural customs [4].

It commonly occurs on the right side and the ventrolateral aspect of the proximal third of the penis [6].

In our study, in line with the literature, penile fracture was observed on the right side in 55.5% of patients and proximally in 66.6%.

In the meta-analysis conducted by Amer et al. the most common causes of penile fracture were sexual intercourse (46%), forced flexion (21%), and masturbation (18%) [7].

The most common cause of penile fracture is sexual intercourse in America andWestern European countries, while it is manually bending the penis for detumescence in the Middle East, Gulf region and North Africa [[8], [9], [10], [11]].

In our research, five patients (55.5%) had a penile fracture during sexual intercourse, three patients (33.3%) during masturbation, and one patient (11.1%) had penile fracture due to sudden forced flexion.

In a study by Mahapatra et al., 95% of cases were diagnosed through proper history and physical examination [12]. In a study by Kumar et al., 85% of patients had undergone immediate surgical exploration depending on history and examination findings [13].

In our center, 100% of patients were diagnosed clinically. Then, we decided to move to surgical exploration after just physical examination. In all of our patients, we did not request any type of radiological images (US, MRI).

In Kumar et al. penile fracture associated with urethral injury was found in 15% of patients [13].

In Mahapatra et al. 10% of patients had associated urethral injury with penile fracture [12].

In our case, we did not have patients with penile fracture accompanied with urethral injury. All cases were free from urethral injury.

Penile fracture most commonly occurs on venterolateral aspect of the proximal part of the penis and on the right side. In Kumar et al. most of the tear involved the proximal part of the penis [4].

In our hospital, the most common side for penile fracture was the right side (55.5%). The most common location for penile fracture was proximal shaft (66.6%).

Felter and Gartmen in 1936 first described the surgical repair of penile fracture.

In Kumar et al., 90% of patients were explored immediately who had good outcomes [13].

Our patients underwent surgical exploration within 6 h of presentation. We had not any patient who was treated conservatively.

El Atat et al. concluded that early surgical repair was associated with low frequency of complications [14]. In another study, Bozzini et al. concluded that surgical repair performed within the first 8.23 h reduced the risk of erectile dysfunction after surgery [15].

One limit of our study is that patients were not followed-up. This is because of Syrian crisis and bad circumstances.

## 5 Conclusion

Patients with penile fracture should be managed as soon as possible to avoid complications. Most cases can be diagnosed without radiological imaging. Surgical exploration with absorbable sutures is the most common treatment.

## Provenance and peer review

Not commissioned, externally peer-reviewed.

## Please state any conflicts of interest

All authors disclose any conflicts of interest.

## Please state any sources of funding for your research

We do not have any financial sources for our research.

## Ethical approval

The article is exempted from ethical approval.

## Consent

N/A, a retrospective analysis of medical records.

## Author contribution

Maher Al-Hajjaj: contributed in study concept and design, data collection, and writing the paper. Ali Alali Aljool: contributed in data interpretation and writing the paper Hassan Al Husien: Helped in writing and reviewing the manuscript.

## Registration of research studies

1. Name of the registry: OSF Preregistration.
2. Unique Identifying number or registration ID: osf. io/wsbdf
3. Hyperlink to your specific registration (must be publicly accessible and will be checked): https://archive.org/details/osf-registrations-fec78-v1

## Guarantor

Maher Al-Hajjaj.
